# Decisional Conflict and Patient Experiences in Dialysis Treatment Decision-Making: A Mixed-Methods Study in a Portuguese Cohort

**DOI:** 10.3390/healthcare14070900

**Published:** 2026-03-31

**Authors:** Ingrid Bispo, Francisca Rego, Guilhermina Rego

**Affiliations:** RISE-Health, Faculty of Medicine, University of Porto, Alameda Prof. Hernâni Monteiro, 4200-319 Porto, Portugal; mfrego@med.up.pt (F.R.); guilherminarego@med.up.pt (G.R.)

**Keywords:** decisional conflict, dialysis decision-making, kidney failure, shared decision-making, patient engagement, patient-centered care, psychosocial adaptation, coping

## Abstract

**Highlights:**

**What are the main findings?**

- Decisional conflict among patients initiating dialysis was generally low, but a substantial proportion of participants still reported uncertainty regarding the implementation of their decision.
- Qualitative findings revealed limited engagement in decision-making, a perceived absence of choice, and a process of resignation and coping as patients adapted to dialysis initiation.

**What are the implications of the main findings?**

- Decisional conflict in kidney failure care is influenced not only by information availability but also by patients’ perceived agency, communication dynamics, and psychosocial adaptation to chronic illness.
- Patient-centered communication and structured opportunities to explore personal values early in the disease trajectory may help reduce uncertainty and support more meaningful engagement in treatment decision-making.

**Abstract:**

**Background/Objectives**: Choosing a treatment modality for kidney failure represents one of the most complex decisions faced by patients with advanced chronic kidney disease. Decisional conflict may arise when patients feel uncertain, insufficiently informed, or unclear about their personal values, potentially affecting treatment satisfaction and long-term adaptation. This study examined decisional conflict among patients initiating dialysis and explored how patients described their experiences during the decision-making process. **Methods**: This exploratory mixed-methods study was conducted at a university-affiliated hospital in Portugal and included 32 adults initiating dialysis following structured pre-treatment education about kidney failure treatment options. Decisional conflict was assessed using the Decisional Conflict Scale, and sociodemographic data were collected. Participants’ reflections expressed during questionnaire administration were documented verbatim and evaluated using thematic analysis. Quantitative data were analyzed using non-parametric statistics. **Results:** Overall decisional conflict levels were low, although the uncertainty subscale showed a moderate mean score. Twenty-five percent of participants reported moderate to high uncertainty regarding decision implementation. Age was positively associated with the “informed” subscale, suggesting that older participants reported greater difficulty feeling adequately informed during decision-making. Qualitative findings identified three themes: lack of engagement in decision-making, perceived absence of choice, and resignation and coping. These themes reflected the influence of clinician authority, the perception of dialysis as an inevitable life-preserving intervention, and patients’ emotional adaptation to treatment. **Conclusions**: Decisional conflict during dialysis initiation appears to be shaped by the interplay between information exchange, values clarification, and perceived autonomy. These findings highlight the importance of patient-centered communication strategies that support deliberation and meaningful engagement in treatment decisions.

## 1. Introduction

Kidney failure is a growing global health challenge, associated with high morbidity, mortality, and diminished quality of life [1,2]. Worldwide, the number of individuals requiring kidney replacement therapy (KRT) continues to increase, driven by population aging and the rising prevalence of diabetes, hypertension, and cardiovascular disease [3]. As life expectancy rises, an increasing proportion of patients initiating dialysis are older adults, making kidney failure increasingly prevalent among individuals aged 65 years and above [4].

Portugal is among the European countries with one of the highest incidence rates of patients receiving KRT [5]. National registry data indicate that approximately 61% of patients initiating dialysis are male [5]. The median age at initiation is close to 68 years for hemodialysis (HD) and 53 years for peritoneal dialysis (PD) [5]. HD remains the predominant modality at treatment initiation, accounting for over 80% of cases, while PD is less frequent [5]. Only 12% of individuals with stage 5 of chronic kidney disease (CKD) are referred to conservative kidney care, and more than 95% of transplant patients undergo a period of dialysis prior to transplantation [5]. Moreover, a considerable proportion of patients are referred late to nephrology services, potentially limiting opportunities for structured pre-dialysis education and shared decision-making [6]. These epidemiological and organizational characteristics provide important contextual grounding for understanding how dialysis decisions are experienced within the Portuguese healthcare system.

As the prevalence of kidney failure increases, patients must make a critical decision regarding their treatment pathway, whether through HD, PD, kidney transplantation, or conservative care [7]. This choice is inherently complex, as each option has different impacts on daily routines, patient autonomy, and quality of life [8]. Choosing a treatment pathway, therefore, represents not only a technical decision but also a deeply personal one, shaped by individual values, expectations, and social context [9].

Despite the availability of multiple treatment options, many patients report feeling inadequately prepared for this decision, with limited understanding of available options and insufficient support during the decision-making process [10,11,12]. In some settings, patient involvement is limited by medical paternalism, a model of care in which clinicians make decisions on behalf of patients based on what they judge to be in the patient’s best interest, thereby leaving individuals passive in a process that profoundly affects their lives [13]. Such dynamics may contribute to decisional conflict, defined as the uncertainty or difficulty patients experience when weighing medical options, often arising from insufficient information, unclear personal values, limited support, or concerns about risks and outcomes [14]. The prevalence of decisional conflict among patients with kidney failure varies across different cultural, social, and demographic contexts [15,16,17,18]. Higher levels of decisional conflict have been associated with psychological distress, lower satisfaction with treatment, reduced adherence, and, in some cases, decisional regret [19,20]. Decisional conflict may also reflect broader psychological challenges faced by patients with chronic illness, including uncertainty, emotional distress, and difficulties adapting to life-changing treatments [19]. Within this context, the clarification of personal values, such as priorities related to quality of life, independence, symptom burden, and longevity, has been recognized as a key component of shared decision-making and an important factor in reducing decisional conflict [20,21].

Although previous studies have examined determinants of decisional conflict in KRT decision-making, findings remain inconsistent and are often focused on quantitative outcomes, leaving important questions regarding how patients experience and interpret the decision-making process in different healthcare and cultural contexts [21]. Multiple factors contribute to its emergence. Although clinical guidelines advocate the use of decision aids to support decision-making among patients with kidney failure, substantial barriers persist in creating a genuine dialogue between healthcare professionals and patients [22,23]. Many nephrologists report discomfort discussing kidney disease trajectories or end-of-life issues, fearing that such conversations might diminish patients’ hope [24,25,26]. In addition, patients’ difficulty in articulating or clarifying personal values is a persistent barrier that requires deliberate, timely, and empathetic exploration during consultations [27]. Treatment decisions are also often insufficiently individualized, failing to account for patients’ diverse ages, cultural backgrounds, social circumstances, and personal priorities [4,28,29]. Notably, mixed-methods evidence examining decisional conflict in KRT decision-making remains limited in Southern European contexts, including Portugal, where relational understandings of autonomy and traditionally strong physician authority may shape decision-making dynamics differently from those described in North American, Asian, or other European settings, often resulting in patients assuming a more passive role in the decision-making process [30].

This mixed-methods study, therefore, aimed to (a) quantitatively assess decisional conflict among patients starting KRT within a Portuguese cohort and examine factors related to its emergence and (b) qualitatively explore how patients describe autonomy and agency when making decisions about KRT in this cultural and healthcare context. By integrating quantitative and qualitative findings through a mixed-methods framework, the study seeks to deepen understanding of patients’ experiences of decision-making in kidney care and to inform more patient-centered approaches to shared decision-making.

## 2. Materials and Methods

### 2.1. Study Design and Participants

This is an exploratory, embedded mixed-methods study that aimed to generate insights about the decision-making process regarding KRT in kidney care. The quantitative component explored the levels of decisional conflict and its associations with sociodemographic characteristics, while the qualitative component sought to analyze how patients described their experiences, emotions, and reasoning during the decision-making process.

The study took place at a university-affiliated hospital in northern Portugal between March 2023 and December 2024. A trained interviewer conducted individual, face-to-face interviews in a private room, scheduled either after dialysis sessions or following nephrology appointments to minimize participant displacement burden. Quantitative and qualitative data were collected concurrently during the same interviews.

Participants were recruited through consecutive convenience sampling during routine nephrology consultations. All eligible patients attending the clinic during the study period were invited to participate. All included participants initiated KRT in a planned, non-urgent setting. They were regularly followed in the nephrology service prior to KRT initiation and underwent a structured education process, including an information consultation that provided written materials about available dialysis modalities and conservative kidney care, as well as individualized explanations from healthcare professionals. Although conservative kidney care was also presented during this education process, none of the eligible patients followed this treatment pathway at the time of recruitment, and therefore, the analytic sample includes only individuals initiating dialysis. Inclusion criteria were individuals aged 18 years or older, diagnosed with kidney failure according to Kidney Disease: Improving Global Outcomes (KDIGO) criteria [31], and within their first month of KRT initiation. Patients with cognitive impairment, visual or hearing impairment, or those who had previously undergone kidney transplantation were excluded. Ethical approval was granted by the hospital’s research ethics committee.

### 2.2. Information to the Participant and Informed Consent

All participants received written information about the study and provided written informed consent prior to participation.

### 2.3. Sociodemographic Data and KRT Modality

Information on age, gender, and educational level was collected from all participants. The type of KRT initiated was also recorded. As none of the participants followed a conservative kidney management pathway at the time of recruitment, only dialysis modalities (HD or PD) were considered in the analysis.

### 2.4. Decisional Conflict Assessment

Decisional conflict was assessed using the Decisional Conflict Scale (DCS), a 16-item validated instrument designed to measure patients’ personal perceptions of uncertainty in healthcare decision-making [32]. The scale comprises five subscales that capture distinct but interrelated dimensions of decisional conflict: (a) uncertainty in choosing among options; (b) informed, reflecting perceived adequacy of information regarding benefits and risks; (c) value clarity, assessing the extent to which patients feel clear about their personal values related to the decision; (d) support, evaluating perceived support and guidance during decision-making; and (e) effective decision, measuring confidence that the choice was informed, value-based, and satisfactory. Items are rated on a 5-point Likert scale ranging from 0 (strongly agree) to 4 (strongly disagree). Subscale scores and the total score are calculated by averaging item responses and multiplying by 25, yielding standardized scores ranging from 0 to 100. Higher scores indicate greater decisional conflict and should be interpreted as follows: greater uncertainty regarding the decision made (uncertainty subscale), lower perception of feeling informed (informed subscale), reduced clarity regarding personal values (value clarity subscale), lower perceived support in the decision (support subscale), and poor effective decision (effective decision subscale) [32]. Consistent with prior literature, scores below 25 are generally associated with readiness to implement decisions, whereas scores above 37.5 suggest decision delay or persistent uncertainty [33]. A validated Portuguese version of the DCS was used in this study [34]. The internal consistency of the DCS and subscales was assessed using Cronbach’s alpha.

### 2.5. Quantitative Data Analysis

Given the exploratory mixed-methods nature of this study, the main objective was to describe patterns and generate preliminary, hypothesis-generating insights rather than to establish predictive or causal models. Consequently, a formal sample size was not calculated. The final sample consisted of 32 participants, representing all eligible individuals attending the nephrology service during the recruitment period who met the inclusion criteria. This sample size is consistent with methodological recommendations for exploratory designs, where the focus is on depth of understanding, variability of responses, and feasibility within a clinical context.

Due to the small sample size and the non-normal distribution of continuous variables, non-parametric statistical methods were employed. Given the exploratory design and limited sample size, more complex multivariable modeling approaches, such as regression models, were not performed, as they could not provide reliable or stable estimates in this context. Spearman’s rank correlation coefficients were calculated to examine associations between age and DCS scores. In addition, correlations among DCS subscales were explored to better understand how the different dimensions of decisional conflict (uncertainty, informed, value clarity, support, and effective decision) interact within this specific population. Given the multidimensional structure of the DCS, this exploratory analysis allowed for the examination of the relationships between distinct but related decisional domains in the context of dialysis initiation. Between-group differences in DCS scores across dichotomous variables (gender, educational level, and dialysis modality) were evaluated using the Mann–Whitney U test. Statistical significance was set at *p* < 0.05. All analyses were performed using IBM SPSS Statistics (version 29.0.2.0).

### 2.6. Qualitative Data Collection and Analysis

Although the interview process was structured around the DCS items, participants were encouraged to elaborate on their reasoning, perceptions, and experiences related to KRT decision-making. In particular, the statement “This decision is easy for me to make” (item 12 of the DCS) often served as a prompt for participants to further reflect on their experiences and emotions regarding the decision-making process. Participants’ statements regarding their lived experiences through the decision-making process were documented verbatim during the interview and immediately transcribed after participants’ responses to preserve the accuracy of expression. These spontaneous reflections were retained, as they capture participants’ immediate perceptions and emotional responses to the decision-making process, thereby providing contextual insight that complements the quantitative findings. This approach generated qualitative data through participants’ reflections expressed during questionnaire administration, consistent with an embedded mixed-methods design [35].

Data were analyzed after the removal of personal identifiers to ensure participant confidentiality. The qualitative evaluation followed Braun and Clarke’s six-phase framework for thematic analysis: (1) familiarization with the transcripts through repeated reading; (2) generation of initial codes capturing key concepts; (3) organization of codes into potential themes; (4) review and refinement of themes for coherence and distinctiveness; (5) definition and naming of themes; and (6) selection of illustrative quotations [36]. Coding was conducted to identify patterns emerging from participants’ narratives, while interpretation remained informed by the conceptual dimensions of decisional conflict represented in the DCS (uncertainty, informed, value clarity, support, and effective decision). This hybrid analytic approach enabled the exploration of experiential meanings underlying decisional conflict while maintaining conceptual coherence with the established domains of the scale.

An initial set of three patients’ statements was independently coded by two researchers (I.B. and F.R.) to develop a preliminary codebook. Coding was performed without the use of qualitative analysis software. Interrater reliability was assessed using Krippendorff’s alpha to ensure coding consistency. Discrepancies were discussed and resolved through consensus, and the remaining data were subsequently coded using the agreed-upon framework. Identified themes were cross-checked against the original transcripts to ensure consistency and fidelity to participants’ narratives. Themes were refined iteratively through ongoing discussion between researchers until conceptual saturation was reached.

Integration between the quantitative and qualitative components occurred during interpretation, whereby qualitative themes were used to contextualize and provide explanatory insight into the patterns observed in the quantitative analysis. Reporting of the qualitative component followed the Consolidated Criteria for Reporting Qualitative Research (COREQ) guidelines to ensure methodological transparency and rigor [37].

## 3. Results

### 3.1. Sociodemographic Characteristics

Of the 44 patients invited to participate, 32 were included in the final sample. Twelve patients were not included because of the following reasons: six patients refused to participate, two of them had cognitive impairment, two patients had hearing impairment, one patient had visual impairment, and one patient had previously undergone kidney transplantation. The mean age was 59 years. Most participants were male (69%), and 59% (n = 19) had fewer than 12 years of formal education. Approximately half of the patients chose peritoneal dialysis (53%) as treatment for kidney failure. Detailed sociodemographic and clinical characteristics are presented in Table 1.

### 3.2. DCS Total Score and Subdomains

The DCS total score demonstrated good internal consistency in this sample (Cronbach’s α = 0.86). However, subscale reliability varied, with lower values (α < 0.6) observed for the “uncertainty,” “support,” and “effective decision” subscales. The “informed” and “value clarity” subdomains demonstrated moderate to good reliability (α = 0.64 and α = 0.85, respectively). These findings should be interpreted with caution, particularly for subscales with lower internal consistency. All Cronbach’s α values are reported in Table 2.

Using a cut-off score of 25 to indicate readiness to implement a decision, 24 participants (75%) were classified as able to implement their decision, whereas 8 participants (25%) reported moderate or substantial uncertainty regarding decision implementation. Overall, mean scores for the DCS total and its subscales were low, with the exception of the uncertainty subdomain, which demonstrated a moderate mean score (26.3 ± 20.9). Correlations among the DCS subscales ranged from moderate to strong. Higher uncertainty was associated with lower perceived information (ρ = 0.596), reduced clarity about personal values (ρ = 0.407), lower perceived support (ρ = 0.558), and lower confidence in the decision made (ρ = 0.489). Lower perceived information was also associated with reduced value clarity (ρ = 0.618) and lower perceived support (ρ = 0.658). Similarly, reduced value clarity was moderately associated with lower perceived support (ρ = 0.458) and lower confidence in the effectiveness of the decision (ρ = 0.569). In contrast, perceived support showed no significant association with the effective decision subscale (ρ = 0.273) (Table 3).

### 3.3. DCS and Sociodemographic Data

**Figure 1:**
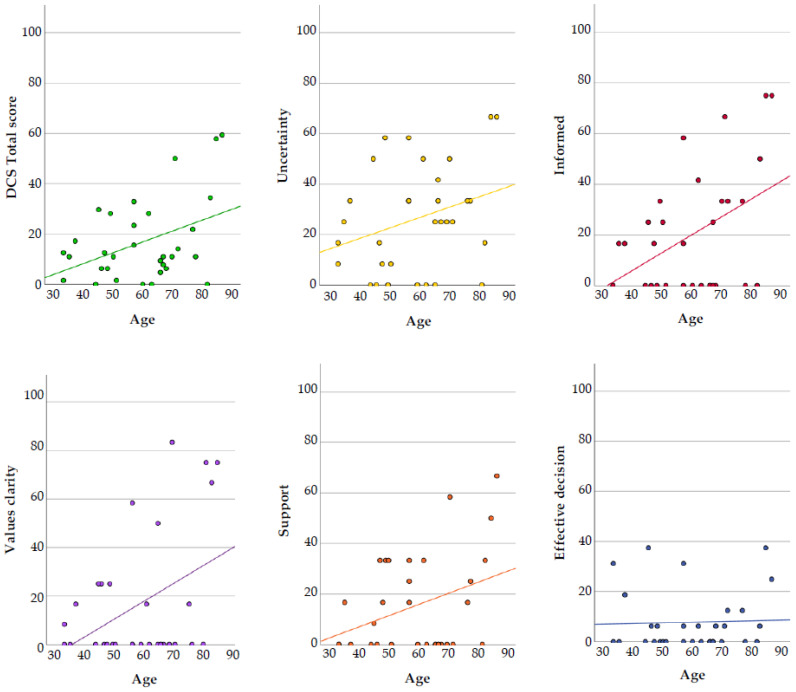
Scatterplots illustrating associations between age and Decisional Conflict Scale (DCS) total score and subscales. DCS scores range from 0 to 100, and age is presented in years. Lines represent fitted trends; plots are presented for descriptive purposes.

No statistically significant differences in total DCS scores were found across gender, educational level, or dialysis modality (Supplementary Materials). Table 4 presents Spearman’s rank correlation between age and DCS scores. Age showed a non-significant, although positive, correlation with the DCS total score (ρ = 0.25, *p* = 0.164). A statistically significant positive association was found between age and the “informed” subscale (ρ = 0.38, *p* = 0.033), suggesting that older participants reported greater difficulty feeling adequately informed during the decision-making process. Non-significant, although positive, correlations were also observed between age and the “uncertainty” (ρ = 0.29, *p* = 0.111), “value clarity” (ρ = 0.21, *p* = 0.259), and “support” (ρ = 0.24, *p* = 0.194) subscales. No meaningful association was identified between age and the “effective decision” subscale (ρ = 0.10, *p* = 0.594). Figure 1 illustrates the direction of these associations for descriptive purposes.

### 3.4. Qualitative Data

Three themes emerged from the thematic analysis: (1) lack of engagement in decision-making, referring to participants’ accounts of delegating the decision to clinicians or being strongly influenced by others during the decision process; (2) perceived absence of choice, describing situations in which dialysis was framed as a “life-or-death” necessity rather than a deliberative option; and (3) resignation and coping, reflecting expressions of acceptance of the disease and adaptation to dialysis as an unavoidable reality. Interrater reliability between coders was high (Krippendorff’s α > 0.80), indicating strong agreement in the coding process. Illustrative excerpts from participants’ comments are presented in Table 5. The first two themes were predominant, with many participants describing the decision as largely determined by medical recommendation or clinical necessity, often accompanied by feelings of limited agency and inevitability. The third theme captured participants’ adaptive responses, characterized by gradual acceptance, cognitive reframing, and emotional adjustment to dialysis.

## 4. Discussion

This study examined decisional conflict among patients initiating KRT and explored how patients described their experiences during the treatment decision-making process. Quantitative and qualitative findings converged in suggesting that decisional conflict during dialysis initiation is shaped not only by the availability of information but also by how patients experience their role within the decision-making process. Although overall levels of decisional conflict were low in this sample, a substantial proportion of participants still reported uncertainty regarding the implementation of their decision. The qualitative findings further revealed that many patients perceived limited engagement in decision-making, experienced dialysis as an unavoidable necessity, and described a process of emotional adaptation to treatment.

Overall, levels of decisional conflict in this sample were low, although the uncertainty subscale showed a moderate mean score. Notably, 25% of participants still reported moderate to high uncertainty regarding the implementation of their decision. Correlations among the DCS subscales suggest that different dimensions of decisional conflict interact during KRT decision-making: participants who reported lower perceived information also tended to report lower clarity regarding their personal values, and both domains were associated with higher levels of uncertainty. Previous studies have noted that insufficient or poorly timed information about disease prognosis and treatment pathways in advanced kidney disease may contribute to lower treatment adherence and poorer quality of life [38,39]. Although effective communication about these issues remains challenging for clinicians [10,40], patients frequently express a preference for clear and timely information about disease trajectories and treatment implications [25,41]. When communication is limited or cautiously framed, decision-making may become more clinician-directed, reducing opportunities to explore patients’ personal values and potentially resulting in treatment choices that are not fully aligned with patients’ preferences, which may contribute to later regret [20,42]. The patterns observed in the present study, therefore, suggest that uncertainty may function as a sensitive indicator of tensions within the decision-making process, particularly when information, support, and personal value clarification are not fully integrated during clinical discussions [43]. These findings should, however, be interpreted with caution, as variability in subscale reliability may have influenced the strength and direction of the observed associations.

The DCS “informed” subscale also appeared to be associated with age in this cohort, suggesting that older participants may experience greater difficulty feeling adequately informed during the decision-making process. For older and frail patients, communicating prognostic uncertainty and treatment trajectories in advanced kidney disease remains particularly challenging, as dialysis may not consistently provide clear survival or quality of life benefits [44]. In clinical practice, discussions about conservative management or end-of-life care remain difficult and are sometimes limited due to concerns about diminishing patients’ hope [45]. As a result, dialysis may be implicitly framed as the default life-preserving option, which can narrow the perceived space for deliberation [43]. Within such contexts, older patients may be more likely to defer to medical authority, experiencing the decision less as an active choice and more as an inevitable clinical pathway [46].

The qualitative findings provide additional context for these quantitative patterns. The theme of lack of engagement in decision-making reflects a subtle yet powerful tension between medical authority and patient agency that may arise when treatment decisions are strongly guided by clinical recommendations [13]. Participants frequently described delegating decisions to clinicians or relying heavily on medical advice, suggesting that the decision-making process was often experienced as clinician-led rather than jointly deliberative. This pattern may partly reflect structural and cultural characteristics of healthcare decision-making in Portugal, where studies have reported a relatively strong role of physician authority and a tendency for patients to adopt a more passive role in medical decisions [30]. This pattern aligns with the quantitative findings that indicate that lower perceived information and reduced value clarity were associated with greater uncertainty. Previous studies have similarly reported that some patients initiate dialysis partly to please their physicians, to fulfill the role of being a “good patient,” or under the influence of family expectations, circumstances that may reduce long-term treatment satisfaction [4,47,48,49]. Such dynamics may be particularly evident in clinical contexts where medical authority is perceived as the “final word” or where family preferences overshadow individual deliberation [50]. In these situations, decision-making may be only partially experienced as autonomous, limiting patients’ sense of agency even when clinical information is provided [4,49,51,52].

Closely related to this dynamic, the theme of the perceived absence of choice illustrates how dialysis initiation was often experienced as an unavoidable necessity rather than as an active decision. Several participants described dialysis as the only viable option for survival, transforming a medical recommendation into a perceived moral imperative in which declining treatment could be equated with refusing life itself [26,43,53]. This framing has been widely reported in nephrology literature and has been associated with limited exploration of alternative pathways such as conservative kidney care, particularly for older or frail patients [4,24]. National registry data from Portugal further illustrate this pattern, showing that only a small proportion of patients transition from dialysis to conservative kidney care based on patient preference, while the majority of such decisions are primarily guided by clinicians [5]. Communication plays a central role in this process. When dialysis is presented primarily as a life-preserving intervention, there may be limited opportunities to align treatment choices with patients’ expectations regarding daily routines and quality of life, both of which are substantially affected by dialysis [9,20,27]. When information is limited or the burdens and side effects of dialysis are minimized, patients may develop unrealistic expectations or fail to fully understand the implications of different treatment pathways, a pattern that has also been associated with later decisional regret [20,23,24,26,54]. At the same time, patients consistently report a desire for clear, honest, and timely communication about disease trajectories and treatment consequences [24,25]. Within this communicative tension, the perception of limited choice may become intertwined with insufficient exploration of personal values, reinforcing the quantitative finding that lower perceived information and reduced value clarity were associated with higher uncertainty in the present study [20]. For older adults, who are already facing physical decline and increased vulnerability, this dynamic may create a subtle form of existential coercion, where agreement with dialysis reflects necessity rather than genuine preference [44,55,56]. Thus, what appears externally as acceptance may internally coexist with underlying uncertainty, underscoring the ethical importance of progressive, empathetic, and patient-tailored communication that allows individuals the time and space to reflect, seek support, and align decisions with their values [57,58,59].

Finally, the theme of resignation and coping captures how patients emotionally adapt to these circumstances [60]. Many participants described a gradual shift from initial resistance or fear toward acceptance of dialysis as an unavoidable aspect of disease progression. This form of acceptance may represent a way of emotional coping, helping individuals restore coherence and meaning in the face of chronic illness [59,61,62]. For several participants, particularly older individuals, coping involved trusting clinicians’ judgment and adapting to treatment rather than actively shaping the decision [63,64]. This adaptive response may help explain the relatively low levels of decisional conflict observed in the present sample. Acceptance of the disease and trust in medical guidance may reduce the subjective experience of conflict even when deliberative engagement in the decision-making process remains limited [38,65]. Patients may require different periods of time to process the implications of kidney failure and to develop acceptance of treatment [66]. These variations highlight the importance of initiating discussions about treatment planning early in the disease trajectory, allowing sufficient time to explore patients’ values and expectations before urgent decisions become necessary while also creating opportunities to revisit decisions as circumstances evolve [66,67]. In this sense, coping can be understood not only as an adaptive strategy but also as a psychosocial process through which patients seek to preserve dignity and continuity of identity in the face of disease progression within a clinical context where decisions may be experienced as constrained [26].

This study has several limitations that should be considered when interpreting the findings. First, the sample size was modest and derived from a single center, which may limit the generalizability of the findings and the robustness of the statistical analyses. The exploratory nature of the study and the limited sample size also precluded the use of more complex statistical approaches, such as multivariate regression models. Therefore, the quantitative findings should be interpreted as hypothesis-generating rather than confirmatory, and future studies with larger samples are warranted to enable more comprehensive modeling of factors associated with decisional conflict and to further examine relationships between clinical, demographic, and psychosocial variables. Second, although the DCS demonstrated good internal consistency at the total-score level, some subscales showed low reliability (Cronbach’s α < 0.60). As such, findings related to individual subdomains should be interpreted with caution, as these measures may not fully capture the intended constructs and may have influenced the observed associations [64,68]. Third, although conservative kidney care was discussed during pre-dialysis education, no participants had followed this pathway at the time of recruitment, which limited the ability to explore decisional conflict in patients considering non-dialytic management. Fourth, the qualitative data were generated within questionnaire administration rather than through in-depth interviews, which may have limited the depth and richness of the narratives, as in-depth interviews would allow for more comprehensive exploration of participants’ personal reflections and facilitate a more nuanced and interpretative thematic analysis. Fifth, decisional conflict may evolve over time as patients adapt to daily routines, gradually clarify their personal values, and align treatment expectations with lived experience. As this study captured perceptions during the early phase of treatment initiation, it may not fully reflect how decisional conflict develops over time. Finally, further research is needed to explore how symptom burden, mental health, and overall quality of life influence decisional conflict, as these factors may shape patients’ experiences and engagement in the decision-making process [55,69,70,71].

## 5. Conclusions

In this study, decisional conflict at the initiation of dialysis appeared to be associated with age and with how patients experienced the decision-making process. Older adults reported greater difficulty feeling adequately informed during decision-making. The qualitative findings further illuminated the emotional and relational dynamics underlying this conflict: many participants described a lack of engagement in the decision-making process, a perceived absence of choice and a sense of inevitability, and a trajectory of resignation and coping as they adapted to what was often framed as an unavoidable, life-preserving therapy. These patterns highlight how decisional conflict is not merely a reflection of information deficits but is also shaped by how patients interpret the necessity of dialysis, the influence of clinicians, and the limits of their perceived agency. These insights underscore the need for patient-centered communication strategies that promote autonomy, explore personal values, and encourage meaningful engagement in treatment decisions, particularly for older adults, who may be most vulnerable to uncertainty and to paternalistic decision-making dynamics. Future studies with larger samples and longitudinal designs are warranted to confirm these results and to evaluate targeted interventions to reduce decisional conflict in kidney failure care.

## Figures and Tables

**Table 1:** Sociodemographic characteristics of the patients included in the study.

	All Participants (n = 32)
**Mean age**	
mean ± SD	59.7 ± 15.5
**Gender** n (%)	
Female	10 (31%)
Male	22 (69%)
**Educational level** n (%)	
<12 years	19 (59%)
12 years or more	13 (41%)
**Dialysis modality** n (%)	
Hemodialysis	15 (47%)
Peritoneal dialysis	17 (53%)

SD: standard deviation.

**Table 2:** Cronbach’s alpha values of the Decisional Conflict Scale (DCS) total score and subdomains.

	Scale Items	Cronbach’s Alpha
Total score	all	0.86
Uncertainty	10, 11, 12	0.46
Informed	1, 2, 3	0.64
Value clarity	4, 5, 6	0.85
Support	7, 8, 9	0.30
Effective decision	13, 14, 15, 16	0.54

**Table 3:** Decisional Conflict Scale (DCS) scores and correlations among the scale subdomains.

DCS Subscales	Mean Score ± SD	Correlation Coefficients				
		Uncertainty	Informed	Value Clarity	Support	Effective Decision
Total score	16.7 ± 16.1	___	___	___	___	___
Uncertainty	26.3 ± 20.9	1.000	**0.596** **	**0.407** *	**0.558** **	**0.489** **
Informed	20 ± 24	___	1.000	**0.618** **	**0.658** **	**0.393** *
Value clarity	16.9 ± 26.7	___	___	1.000	**0.458** **	**0.569** **
Support	15.6 ± 19.3	___	___	___	1.000	0.273
Effective decision	7.8 ± 11.8	___	___	___	___	1.000

DCS scores range from 0 to 100, with higher scores indicating greater decisional conflict. Scores ≥ 25 are commonly interpreted as clinically significant decisional conflict. SD: standard deviation. All correlation coefficients are Spearman’s rho; *p* < 0.05 (*); *p* < 0.01 (**). Values of 0.30–0.49 indicate moderate correlations, and values ≥ 0.50 indicate strong correlations.

**Table 4:** Correlations between age and DCS (total score and subscales).

Age		Correlation Coefficients				
	Total Score	Uncertainty	Informed	Value Clarity	Support	Effective Decision
Correlation coefficient	0.25	0.28	**0.37** *	0.25	0.23	0.09

All correlation coefficients are Spearman’s rho; *p* < 0.05 (*). Values of 0.30–0.49 indicate moderate correlations, and values ≥ 0.50 indicate strong correlations.

**Table 5:** Themes and illustrative patients’ quotations.

Themes	Illustrative Excerpts from Patients’ Comments
**Lack of engagement in** **decision-making** Decision influenced by others	“At a certain point, I was forced into it.” (P6299, 50–60 y) “The doctor was the one who decided.” (P1578, 50–60 y) “This decision was not mine; it was made for me.” (P1734, 60–70 y)
**Perceived absence of choice** Dialysis framed as a “life or death” decision	“I had no chance to choose.” (P6544, 80–90 y) “If a person doesn’t do it, what happens? They die!” (P1939, 70–80 y) “If it weren’t for this, I’d already be dead.” (P3218, 70–80 y) “It had to be done.” (P2828, 70–80 y) “I postponed it as long as I could. Until the doctor said I wouldn’t leave here without dialysis.” (P3936, 50–60 y)
**Resignation and coping** Disease acceptance	“I have been preparing my mind.” (P1938, 70–80 y) “At first, I didn’t want to do it. Later, I did.” (P1919, 50–60 y) “What has no remedy must be accepted.” (P6981 60–70 y)

y: years. Note: Illustrative quotations were selected to represent typical expressions of each theme and to reflect the diversity of participants’ experiences.

## Data Availability

The data supporting the findings of this study are available upon reasonable request from the corresponding author, subject to compliance with institutional data-sharing policies and applicable privacy or ethical restrictions.

## Supplementary Materials

The following supporting information can be downloaded at: https://www.mdpi.com/article/10.3390/healthcare14070900/s1, Table S1: Mean scores of the Decisional Conflict Scale (DCS) regarding sociodemographic data and dialysis modality.

## Abbreviations

The following abbreviations are used in this manuscript:

KRT	Kidney replacement therapy
HD	Hemodialysis
PD	Peritoneal dialysis
CKD	Chronic kidney disease
DCS	Decisional Conflict Scale

## References

[1] Turin T.C., Tonelli M., Manns B.J., Ravani P., Ahmed S.B., Hemmelgarn B.R. (2012). Chronic kidney disease and life expectancy. Nephrol. Dial. Transplant..

[2] Sprangers B., Van der Veen A., Hamaker M.E., Rostoft S., Latcha S., Lichtman S.M., de Moor B., Wildiers H. (2021). Initiation and termination of dialysis in older patients with advanced cancer: Providing guidance in a complicated situation. Lancet Healthy Longev..

[3] Davison S.N. (2010). End-of-life care preferences and needs: Perceptions of patients with chronic kidney disease. Clin. J. Am. Soc. Nephrol..

[4] Ladin K., Lin N., Hahn E., Zhang G., Koch-Weser S., Weiner D.E. (2017). Engagement in decision-making and patient satisfaction: A qualitative study of older patients’ perceptions of dialysis initiation and modality decisions. Nephrol. Dial. Transplant..

[5] https://www.spnefro.pt/sociedade/gabinete-de-registo-de-doenca-renal-terminal.

[6] Duarte R., Trigo A., Luz I., Santos P., Lopes K., Gonçalves H., Sofia F., Vila Lobos A. (2022). Low income is associated with late nephrology referral in Portugal: A retrospective study. Nephrol. Dial. Transplant..

[7] O’Connor N.R., Kumar P. (2012). Conservative management of end-stage renal disease without dialysis: A systematic review. J. Palliat. Med..

[8] Chiou C.P., Chung Y.C. (2012). Effectiveness of multimedia interactive patient education on knowledge, uncertainty and decision-making in patients with end-stage renal disease. J. Clin. Nurs..

[9] Pawar A.S., Thorsteinsdottir B., Whitman S., Pine K., Lee A., Espinoza Suarez N.R., Lee P.O., Thota A., Lorenz E., Beck A. (2024). Decisional Regret Surrounding Dialysis Initiation: A Comparative Analysis. Kidney Med..

[10] Song M.K., Lin F.C., Gilet C.A., Arnold R.M., Bridgman J.C., Ward S.E. (2013). Patient perspectives on informed decision-making surrounding dialysis initiation. Nephrol. Dial. Transplant..

[11] Goh Z.Z.S., Chia J.M.X., Seow T.Y., Choo J.C.J., Foo M., Seow P.S., Griva K. (2022). Treatment-related decisional conflict in pre-dialysis chronic kidney disease patients in Singapore: Prevalence and determinants. Br. J. Health Psychol..

[12] Ladin K., Pandya R., Perrone R.D., Meyer K.B., Kannam A., Loke R., Oskoui T., Weiner D.E., Wong J.B. (2018). Characterizing Approaches to Dialysis Decision Making with Older Adults: A Qualitative Study of Nephrologists. Clin. J. Am. Soc. Nephrol..

[13] Saeed F., Sardar M., Rasheed K., Naseer R., Epstein R.M., Davison S.N., Mujtaba M., Fiscella K.A. (2020). Dialysis Decision Making and Preferences for End-of-Life Care: Perspectives of Pakistani Patients Receiving Maintenance Dialysis. J. Pain. Symptom Manag..

[14] Stacey D., Legare F., Boland L., Lewis K.B., Loiselle M.C., Hoefel L., Garvelink M., O’connor A. (2020). 20th Anniversary Ottawa Decision Support Framework: Part 3 Overview of Systematic Reviews and Updated Framework. Med. Decis. Mak..

[15] DePasquale N., Green J.A., Ephraim P.L., Morton S., Peskoe S.B., Davenport C.A., Mohottige D., McElroy L., Strigo T.S., Hill-Briggs F. (2022). Decisional Conflict About Kidney Failure Treatment Modalities Among Adults With Advanced CKD. Kidney Med..

[16] Vélez-Bermúdez M., Christensen A.J., Kinner E.M., Roche A.I., Fraer M. (2019). Exploring the Relationship Between Patient Activation, Treatment Satisfaction, and Decisional Conflict in Patients Approaching End-Stage Renal Disease. Ann. Behav. Med..

[17] McPherson L., Basu M., Gander J., Pastan S.O., Mohan S., Wolf M.S., Chiles M., Russell A., Lipford K., Patzer R.E. (2017). Decisional conflict between treatment options among end-stage renal disease patients evaluated for kidney transplantation. Clin. Transplant..

[18] Noble H., Brazil K., Burns A., Hallahan S., Normand C., Roderick P., Thompson C., Maxwell P., Yaqoob M. (2017). Clinician views of patient decisional conflict when deciding between dialysis and conservative management: Qualitative findings from the PAlliative Care in chronic Kidney diSease (PACKS) study. Palliat. Med..

[19] Tan E.G.F., Teo I., Finkelstein E.A., Meng C.C. (2019). Determinants of regret in elderly dialysis patients. Nephrology.

[20] Bispo I., Bressan M., Rego F., Rego G. (2025). Decisional regret in adults facing treatment choices for kidney failure: A systematic review. Nephron.

[21] Subramanian L., Zhao J., Zee J., Tentori F. (2018). PCORI Final Research Reports. Does an Online Decision Aid Help People with Advanced Chronic Kidney Disease Choose Between Two Treatment Options?.

[22] Murray M.A., Brunier G., Chung J.O., Craig L.A., Mills C., Thomas A., Stacey D. (2009). A systematic review of factors influencing decision-making in adults living with chronic kidney disease. Patient Educ. Couns..

[23] Chen N.H., Lin Y.P., Liang S.Y., Tung H.H., Tsay S.L., Wang T.J. (2018). Conflict when making decisions about dialysis modality. J. Clin. Nurs..

[24] Ladin K., Pandya R., Kannam A., Loke R., Oskoui T., Perrone R.D., Meyer K.B., Weiner D.E., Wong J.B. (2018). Discussing Conservative Management With Older Patients With CKD: An Interview Study of Nephrologists. Am. J. Kidney Dis..

[25] Wong S.P.Y., McFarland L.V., Liu C.F., Laundry R.J., Hebert P.L., O’Hare A.M. (2019). Care Practices for Patients With Advanced Kidney Disease Who Forgo Maintenance Dialysis. JAMA Intern. Med..

[26] Schell J.O., Patel U.D., Steinhauser K.E., Ammarell N., Tulsky J.A. (2012). Discussions of the kidney disease trajectory by elderly patients and nephrologists: A qualitative study. Am. J. Kidney Dis..

[27] Rego F., Goncalves F., Moutinho S., Castro L., Nunes R. (2020). The influence of spirituality on decision-making in palliative care outpatients: A cross-sectional study. BMC Palliat. Care.

[28] Tamura M.K., Tan J.C., O’Hare A.M. (2012). Optimizing renal replacement therapy in older adults: A framework for making individualized decisions. Kidney Int..

[29] Bispo I., Rego F., Rêgo G. (2025). Understanding quality of life trajectories in dialysis: The role of sociodemographic factors. Proceedings of the 17th World Conference in Bioethics, Medical Ethics and Health Law, Ljubljana, Slovenia, 24–25 November 2025.

[30] Gregorio M., Teixeira A., Henriques T., Pascoa R., Baptista S., Carvalho R., Martins C. (2021). What role do patients prefer in medical decision-making?: A population-based nationwide cross-sectional study. BMJ Open.

[31] KDIGO (Kidney Disease Improving Global Outcomes). https://kdigo.org/guidelines/ckd-evaluation-and-management/.

[32] O’Connor A.M. (1995). Validation of a decisional conflict scale. Med. Decis. Mak..

[33] O’Connor A. (1993). User Manual—Decisional Conflict Scale. http://www.ohri.ca/decisionaid.

[34] Martinho M.J., da Silva M.M., Angelo M. (2013). Scale of conflict in health care decision-making: An instrument adapted and validated for the Portuguese language. Rev. Esc. Enferm. USP.

[35] Pope C., Ziebland S., Mays N. (2000). Qualitative research in health care. Analysing qualitative data. BMJ.

[36] Ahmed S.K., Mohammed R.A., Nashwan A.J., Ibrahim R.H., Abdalla A.Q., Ameen B.M.M., Khdhir R.M. (2025). Using thematic analysis in qualitative research. J. Med. Surg. Public Health.

[37] Tong A., Sainsbury P., Craig J. (2007). Consolidated criteria for reporting qualitative research (COREQ): A 32-item checklist for interviews and focus groups. Int. J. Qual. Health Care..

[38] Winterbottom A.E., Bekker H.L., Conner M., Mooney A.F. (2012). Patient stories about their dialysis experience biases others’ choices regardless of doctor’s advice: An experimental study. Nephrol. Dial. Transplant..

[39] Hickman R.L., Daly B.J., Lee E. (2012). Decisional conflict and regret: Consequences of surrogate decision making for the chronically critically ill. Appl. Nurs. Res..

[40] Berkhout-Byrne N., Gaasbeek A., Mallat M.J.K., Rabelink T.J., Mooijaart S.P., Dekker F.W., van Buren M. (2017). Regret about the decision to start dialysis: A cross-sectional Dutch national survey. Neth. J. Med..

[41] Stringer S., Baharani J. (2012). Why did I start dialysis? A qualitative study on views and expectations from an elderly cohort of patients with end-stage renal failure starting haemodialysis in the United Kingdom. Int. Urol. Nephrol..

[42] Wachterman M.W., Leveille T., Keating N.L., Simon S.R., Waikar S.S., Bokhour B. (2019). Nephrologists’ emotional burden regarding decision-making about dialysis initiation in older adults: A qualitative study. BMC Nephrol..

[43] Russ A.J., Shim J.K., Kaufman S.R. (2007). The value of “life at any cost”: Talk about stopping kidney dialysis. Soc. Sci. Med..

[44] Murtagh F.E., Marsh J.E., Donohoe P., Ekbal N.J., Sheerin N.S., Harris F.E. (2007). Dialysis or not? A comparative survival study of patients over 75 years with chronic kidney disease stage 5. Nephrol. Dial. Transplant..

[45] Ren Q., Shi Q., Ma T., Wang J., Li Q., Li X. (2019). Quality of life, symptoms, and sleep quality of elderly with end-stage renal disease receiving conservative management: A systematic review. Health Qual. Life Outcomes.

[46] Berkhout-Byrne N.C., Voorend C.G.N., Meuleman Y., Mooijaart S.P., Brunsveld-Reinders A.H., Bos W.J.W., van Buren M. (2024). Nephrology-tailored geriatric assessment as decision-making tool in kidney failure. J. Ren. Care.

[47] Chan K., Wong F.K.Y., Tam S.L., Kwok C.P., Fung Y.P., Wong P.N. (2022). Effectiveness of a brief hope intervention for chronic kidney disease patients on the decisional conflict and quality of life: A pilot randomized controlled trial. BMC Nephrol..

[48] Hussain J.A., Flemming K., Murtagh F.E., Johnson M.J. (2015). Patient and health care professional decision-making to commence and withdraw from renal dialysis: A systematic review of qualitative research. Clin. J. Am. Soc. Nephrol..

[49] Zhang S., Cui J., Liu X., He X., Xu Y. (2024). Structural equation modeling analysis of factors influencing decisional conflict between dialysis modality among end-stage kidney disease patients in Wuhan. BMC Nephrol..

[50] Fan R. (1997). Self-determination vs. family-determination: Two incommensurable principles of autonomy: A report from East Asia. Bioethics.

[51] Verberne W.R., Konijn W.S., Prantl K., Dijkers J., Roskam M.T., van Delden J.J.M., Bos W.J.W. (2019). Older patients’ experiences with a shared decision-making process on choosing dialysis or conservative care for advanced chronic kidney disease: A survey study. BMC Nephrol..

[52] Alsing J.F.L., Hayes Bauer E., Brandt F., Kampmann J.D. (2022). Expectations and Experiences of Patients Recently Initiated to Centre-Based Dialysis Treatment. Healthcare.

[53] Kaufman S.R., Shim J.K., Russ A.J. (2006). Old age, life extension, and the character of medical choice. J. Gerontol. B Psychol. Sci. Soc. Sci..

[54] Raj R., Thiruvengadam S., Ahuja K.D.K., Frandsen M., Jose M. (2019). Discussions during shared decision-making in older adults with advanced renal disease: A scoping review. BMJ Open.

[55] Ramakrishnan C., Widjaja N., Malhotra C., Finkelstein E., Khan B.A., Ozdemir S. (2024). Unravelling complex choices: Multi-stakeholder perceptions on dialysis withdrawal and end-of-life care in kidney disease. BMC Nephrol..

[56] Beckwith H., Thomas N., Adwaney A., App E.M., Gaffney H., Hill P., Moabi D., Prout V., Salisbury E., Webster P. (2022). Gender Differences in Experiences and Expectations of Hemodialysis in a Frail and Seriously Unwell Patient Population. Kidney Int. Rep..

[57] Seah A.S., Tan F., Srinivas S., Wu H.Y., Griva K. (2015). Opting out of dialysis—Exploring patients’ decisions to forego dialysis in favour of conservative non-dialytic management for end-stage renal disease. Health Expect.

[58] Muthalagappan S., Johansson L., Kong W.M., Brown E.A. (2013). Dialysis or conservative care for frail older patients: Ethics of shared decision-making. Nephrol. Dial. Transplant..

[59] Ladin K., Buttafarro K., Hahn E., Koch-Weser S., Weiner D.E. (2018). “End-of-Life Care? I’m not Going to Worry About That Yet.” Health Literacy Gaps and End-of-Life Planning Among Elderly Dialysis Patients. Gerontologist.

[60] Lok P. (1996). Stressors, coping mechanisms and quality of life among dialysis patients in Australia. J. Adv. Nurs..

[61] Harwood L., Locking-Cusolito H., Spittal J., Wilson B., White S. (2005). Preparing for hemodialysis: Patient stressors and responses. Nephrol. Nurs. J..

[62] Joseph-Williams N., Edwards A., Elwyn G. (2011). The importance and complexity of regret in the measurement of ‘good’ decisions: A systematic review and a content analysis of existing assessment instruments. Health Expect..

[63] Wikstol D., Pedersen R., Magelssen M. (2021). Public attitudes and health law in conflict: Somatic vs. mental care, role of next of kin, and the right to refuse treatment and information. BMC Health Serv. Res..

[64] Pozzar R.A., Berry D.L., Hong F. (2019). Item response theory analysis and properties of decisional conflict scales: Findings from two multi-site trials of men with localized prostate cancer. BMC Med. Inform. Decis. Mak..

[65] Davison S.N., Torgunrud C. (2007). The creation of an advance care planning process for patients with ESRD. Am. J. Kidney Dis..

[66] Aguilera-Flórez A.I., Morán-Centeno M.A., Bandera-Álvarez C., Cordero-Guerrero M.J., Robles-del Río I., Fernández-Iban R. (2024). Análisis del grado de satisfacción con la elección de tratamiento renal sustitutivo. Enferm. Nefrol..

[67] Kors J.M., Paternotte E., Martin L., Verhoeven C.J., Schoonmade L., Peerdeman S.M., Kusurkar R.A. (2020). Factors influencing autonomy supportive consultation: A realist review. Patient Educ. Couns..

[68] Linder S.K., Swank P.R., Vernon S.W., Mullen P.D., Morgan R.O., Volk R.J. (2011). Validity of a low literacy version of the Decisional Conflict Scale. Patient Educ. Couns..

[69] Li X., Ji W., Wang D., Xu Y., Zhao X., Liang S. (2025). Kidney supportive care in advanced chronic kidney disease: A qualitative meta-synthesis of healthcare professionals perspectives and attitudes. BMC Nephrol..

[70] Morton R.L., Snelling P., Webster A.C., Rose J., Masterson R., Johnson D.W., Howard K. (2012). Factors influencing patient choice of dialysis versus conservative care to treat end-stage kidney disease. Can. Med. Assoc. J..

[71] Beckwith H.K.S., Adwaney A., Appelbe M., Gaffney H.T., Hill P., Moabi D., Prout V.L., Salisbury E., Webster P., Tomlinson J.A. (2021). Perceptions of Illness Severity, Treatment Goals, and Life Expectancy: The ePISTLE Study. Kidney Int. Rep..

