# Supplementary material for: Decisional Conflict and Patient Experiences in Dialysis Treatment Decision-Making: A Mixed-Methods Study in a Portuguese Cohort
Source: Healthcare (Basel). 2026 Mar 31;14(7):900. doi: 10.3390/healthcare14070900 (PMC13073215; doi:10.3390/healthcare14070900)
Supplement: Supplementary file 1 [file healthcare-14-00900-s001.zip › healthcare-4220241-supplementary.pdf]

Table S1. Mean scores of Decisional Conflict Scale (DCS), regarding sociodemographic data and dialysis modality.

**n = 32**	**Total Score** mean ± SD	**Uncertainty** mean ± SD	**Informed** mean ± SD	**Values clarity** mean ± SD	**Support** mean ± SD	**Effective decision** mean ± SD
**Gender**						
Male (n = 22)	14.41 ± 14.22	20.83 ± 20.20	17.04 ± 22.19	14.77 ± 25.44	15.15 ± 18.30	6.81 ± 10.37
Female (n = 10)	21.87 ± 19.58	38.33 ± 18.08	26.66 ± 27.72	21.66 ± 30.22	16.66 ± 22.56	10.00 ± 15.08
p – value	0.45 [b]	**0.02** [b]	0.36 [b]	0.48 [b]	0.95 [b]	0.49 [b]
**Educational level**						
< 12 years (n = 19)	19.98 ± 19.06	29.82 ± 23.12	25.00 ± 28.19	23.68 ± 32.30	18.85 ± 22.02	6.90 ± 11.57
12 years or more (n = 13)	12.01 ± 9.33	21.15 ± 16.87	12.82 ± 14.27	7.05 ± 10.11	10.89 ± 14.18	9.13 ± 12.65
p – value	0.57 [b]	0.32 [b]	0.36 [b]	0.44 [b]	0.47 [b]	0.54 [b]
**Dialysis modality**						
Hemodialysis (n = 15)	21.14 ± 20.83	25.00 ± 23.14	30.00 ± 28.66	26.66 ± 34.09	21.66 ± 23.10	7.08 ± 10.79
Peritoneal dialysis (n = 17)	12.86 ± 9.54	27.45 ± 19.49	11.27 ± 15.00	8.33 ± 14.12	10.29 ± 13.98	8.45 ± 13.06
p – value *	0.52 [b]	0.62 [b]	0.07 [b]	0.27 [b]	0.20 [b]	0.85 [b]

SD: standard deviation; n: number of participants. [b] Mann Whitney *U* test.
